# A Meteorological Table

**Published:** 1804-10-01

**Authors:** 

**Affiliations:** Brompton


### *A Meteorological Table, by Dr.* Higgins, *of Brompton.*

| Days of the Month. | Thermometer. | | | Height of the Barometer. Inches. | | | Weather. |
|---|---|---|---|---|---|---|---|
| | 8 o'Clock Morning. | Noon. | 10 o'Clock Night. | 8 o'Clock Morning. | Noon. | 10 o'Clock Night. | |
| 1804. | | | | | | | |
| Aug. 20 | 58° | 62° | 60° | 29.98 | 29.96 | 29.96 | Cloudy. |
| 21 | 60 | 62 | 59 | 30.02 | 30.02 | 30.03 | Cloudy. |
| 22 | 58 | 62 | 57 | .09 | .10 | .18 | Cloudy. |
| 23 | 58 | 60 | 57 | .33 | .36 | .36 | Cloudy. |
| 24 | 58 | 62 | 59 | .40 | .35 | .33 | Fair. |
| 25 | 57 | 62 | 60 | .35 | .38 | .41 | Cloudy. |
| 26 | 60 | 64 | 61 | .52 | .49 | .45 | Fair. |
| 27 | 61 | 65 | 62 | .41 | .37 | .29 | Fair. |
| 28 | 63 | 67 | 65 | .32 | .31 | .30 | Fair. |
| 29 | 68 | 72 | 70 | .29 | .24 | .11 | Fair. |
| 30 | 70 | 74 | 70 | 29.96 | 29.94 | 30.00 | Fair. |
| 31 | 71 | 73 | 70 | 30.14 | 30.08 | 30.06 | Fair. |
| Sept. 1 | 68 | 70 | 66 | .11 | .18 | .29 | Fair. |
| 2 | 64 | 66 | 64 | .34 | .35 | .32 | Fair. |
| 3 | 64 | 66 | 63 | .34 | .35 | .36 | Fair. |
| 4 | 65 | 69 | 67 | .46 | .45 | .44 | Fair. |
| 5 | 68 | 70 | 66 | .44 | .38 | .35 | Fair. |
| 6 | 67 | 70 | 65 | .22 | .14 | .03 | Fair. |
| 7 | 66 | 70 | 64 | .04 | .12 | .15 | Fair. |
| 8 | 65 | 70 | 63 | .33 | .32 | .35 | Fair. |
| 9 | 65 | 70 | 62 | .35 | .27 | .26 | Fair. |
| 10 | 63 | 68 | 63 | .12 | .09 | .07 | Cloudy. |
| 11 | 65 | 70 | 64 | .24 | .28 | .33 | Fair. |
| 12 | 66 | 78 | 68 | .32 | .21 | .15 | Fair. |
| 13 | 67 | 76 | 70 | .11 | .09 | .06 | Fair. |
| 14 | 66 | 76 | 70 | .03 | 29.96 | 29.94 | Fair. |
| 15 | 68 | 75 | 69 | 29.99 | .96 | .94 | Fair. |
| 16 | 69 | 78 | 70 | 30.04 | 30.09 | 30.16 | Fair. |
| 17 | 68 | 72 | 64 | .23 | .26 | .29 | Cloudy. |
| 18 | 64 | 66 | 63 | .31 | .34 | .30 | Cloudy. |
| 19 | 63 | 65 | 63 | .27 | .21 | .17 | Fair. |

The monthly communications of Dr. H. will be particularly acceptable to the Editors; but they wish him to state, for the information of their readers who reside at a distance from the Metropolis, the elevation of his house above the river Thames, at Chelsea, and the direction of it on the compass from St. Paul's, that the course of the winds on the respective days may be taken into the account, which will much increase the value of the information.

